# The neuroprotective effects of the combined extract of mulberry fruit and mulberry leaf against hydrogen peroxide-induced cytotoxicity in SH-SY5Y Cells

**DOI:** 10.1186/s12906-023-03930-z

**Published:** 2023-04-13

**Authors:** Nootchanat Mairuae, Nut Palachai, Parinya Noisa

**Affiliations:** 1 Faculty of Medicine, Mahasarakham University, Mahasarakham, 44000 Thailand; 2 School of Biotechnology, Institute of Agricultural Technology, Suranaree University of Technology, Nakhon Ratchasima, 30000 Thailand

**Keywords:** Mulberry fruit, Mulberry leaf, Neuronal cells, SH-SY5Y

## Abstract

The prevalence of dementia is increasing, and most of the causes are related to neuronal cell death. Unfortunately, no effective strategy is available for protecting against this condition. Based on the use of the synergistic concept together with the positive modulation effect of both mulberry fruit and mulberry leaf on dementia, we hypothesized that the combined extract of mulberry fruit and mulberry leaf (MFML) should mitigate neuronal cell death. Neuronal cell damage was induced in SH-SY5Y cells by exposure to hydrogen peroxide at a dose of 200 μM. SH-SY5Y cells were given MFML at doses of 62.5 and 125 μg/mL before induced cytotoxicity. Then, the cell viability was determined *via *MTT assay, and the possible underlying mechanisms were investigated *via* the alterations of superoxide dismutase (SOD), catalase (CAT), glutathione peroxidase (GSH-Px), malondialdehyde (MDA), nuclear factor-κB (NF-κB), and tumor necrosis factor-alpha (TNF-α), together with apoptotic factors including (B-cell lymphoma 2) BCL2, Casapase-3 and Caspase-9. The results showed that MFML significantly enhanced cell viability. It also significantly decreased MDA level, NF-κB, TNF-α, Casapase-3, Caspase-9, but increased SOD, GSH-Px and BCL2. These data demonstrated the neuroprotective effect of MFML. The possible underlying mechanisms might occur partly *via* the improvement of the inappropriate apoptotic mechanisms *via* BCL2, Casapase-3 and Caspase-9 together with the decrease in neurodegeneration induced by the reduction of inflammation and oxidative stress. In conclusion, MFML is a potential neuroprotectant candidate against neuronal cell injury. However, toxicity, animal studies, and clinical trials are essential to confirm these benefits.

**Supplementary Information:**

The online version contains supplementary material available at 10.1186/s12906-023-03930-z.

## Introduction

The prevalence of dementia, a severe neurodegenerative disorder, is rapidly and continuously increasing [1]. It has been demonstrated that more than 55 million people live with dementia worldwide, and there are nearly 10 million new patients every year [2]. In addition, World Health Organization (WHO) has reported that dementia is currently the leading cause of death among all diseases and one of the major causes of disability and dependency among older people globally [2–4]. Moreover, it also has a great impact, not only for people living with dementia, but also for their caregivers, families, and society at large [2, 5]. Unfortunately, there is no specific treatment, and the efficacy of the current therapy is still limited. Therefore, a prevention strategy is required.

Accumulated lines of evidence have revealed that polyphenolic compounds and flavonoids can provide a protective effect against dementia [6–8]. Recently, it has been reported that the phenolic compounds, flavonoids, and anthocyanins extracted from mulberry (*Morus alba* Linn) possess neuroprotective effects against dementia [9, 10].

According to the Ayurvedic and traditional medicine concepts, most of the polyherbal formulations provide better benefit than monoherbal formulations [11]. Polyherbal therapy can approach multi-target sites simultaneously and can provide a synergistic effect giving rise to increasing health benefits [12]. Recent in vitro studies also demonstrate that the combined extracts significantly enhanced the biological activities associated with the pathophysiology of dementia, such as antioxidant and anti-inflammation effects [12–17]. Based on the synergistic effect of the herbal components and the protective effects against neurodegeneration of mulberry fruit and mulberry leaf extracts, it has been hypothesized that the combined extract of mulberry fruit and mulberry leaf should possess better biological activities associated with the pathophysiology of dementia and could improve neuronal cell death in hydrogen peroxide induced SH-SY5Y cytotoxicity. The changes of SOD, CAT, GSH-Px, MDA, NF-κB, and TNF-α together with apoptotic factors including BCL2, Casapase-3 and Caspase-9 were explored to investigate the possible underlying mechanisms.

## Materials and methods

### Preparation of mulberry fruit and mulberry leaf extracts

Mulberry fruit (*Morus alba* Linn. var. Chiangmai) was identified and kindly provided by Mr. Sombat Kongpa, the chief of Queen Sirikit Department of Sericulture Center (Udon Thani Province), Ministry of Agriculture and Cooperatives, Thailand (voucher specimen 61,001 and deposited at the Research Institute of Human High Performance and Health Promotion, Khon Kaen University, Thailand). After identification, both parts of mulberry were cleaned, dried in an oven (Memmert GmbH, USA) at 60 °C for 72 h, and ground to fine powder. Aqueous extract, 50% and 95% hydro-alcoholic extracts were prepared from powder by maceration. Extracts were then subjected to a 10-min centrifugation at 3,000 revolutions per minute (rpm) and filtered with Whatman No. 1 filter paper. The solvent of the filtrate was removed by using a rotary evaporator and freeze dryer (Labconco freeze-drier, Labconco Corporation, Kansas City, MO, USA) [12]. All extracts were used for determination of the contents of polyphenolic compounds and flavonoids, and the biological activities including antioxidants and antiinflammation activities. Extracts which showed the optimal potential of each part were selected to prepare the combined extract of mulberry fruit and mulberry leaf (MFML). The selected extracts were mixed with various concentrations as follows; 50:50, 33:67, 25:75, 20:80, 17:83, 14:86, 86:14, 83:17, 80:20, 75:25, and 67:33 (w/w) of mulberry fruit and mulberry leaf. The optimum ratio was selected for further analyses [12].

### Assessment of total phenolic compounds contents, flavonoid content, and anthocyanin content

Total phenolic compound content in the extracts was measured using the Folin-Ciocalteu colorimetric method in a microplate reader (iMark™ Microplate Absorbance Reader) [18]. In brief, 20 µL of the extract was mixed with the freshly prepared reagent consisting of 20 µL of 50% v/v Folin-Ciocalteu reagent (Sigma-Aldrich, USA) and 158 µL of distilled water and incubated for 8 min. Then, 30 µL of 20% Na_2_CO_3_ (Sigma-Aldrich, USA) was added and incubated at 25 °C in a dark room for 2 h. After incubation, absorbance was measured at 765 nm. Results was expressed as mg gallic acid equivalent (GAE)/mg extract. Various concentrations of gallic acid (Sigma-Aldrich, USA) were used as a standard calibration curve.

Flavonoids content in the extract was determined by using the aluminum chloride method [19]. In brief, 100 µL of the extract at various concentrations were mixed with 100 µL of 2% methanolic aluminum chloride (Sigma-Aldrich, USA) and incubated at 25 °C in a dark room for 30 min. Then, the absorbance at 415 nm was measured against the suitable blank. Results were expressed as µg quercetin equivalent/mg extract. Various concentrations of quercetin (Sigma-Aldrich, USA) were used for preparation of a standard calibration curve.

Anthocyanin content was assessed according to a pH-differential method [20]. In brief, 1 mL of extract was mixed with 2 mL of 0.025 M potassium chloride pH 1.0 and 2 mL of 0.4 M sodium acetate pH 4.5. Then, the mixed solutions were incubated at 25 °C for 10 min after which, the absorbance was measured at 520 and 720 nm using a UV-spectrophotometer (Pharmacia LKB-Biochrom 4060). Anthocyanin content was calculated and expressed as mg of cyanidin-3-glucoside equivalents/mg extract using a molar extinction coefficient (ɛ) of cyanidin-3-O glucoside of 26,900 L mol^−1^ cm^−1^ and molecular weight (MW) (449.2 g mol^−1^).

### Assessment of biological activities

The 1,1-diphenyl-2-picrylhydrazyl radical (DPPH) was used for the measurement of free radical-scavenging activity of the extract. In brief, 0.1 mM methanolic solution of DPPH was prepared and 2 mL of the mixed solution was added to 0.3 mL of various extract concentrations (1–100 mg/mL) and the prepared solution was mixed and incubated for 30 min at 25 °C. After incubation, absorbance at 517 nm was measured against a blank which did not contain the extract using a microplate reader (iMark™ Microplate Absorbance Reader). L-ascorbic acid was selected as a reference standard. The results were expressed in terms of EC_50_ (the concentration in μg/mL required to inhibit radical formation by 50%) [21].

The ability of the extract to convert ferric tripyridyltriazine (Fe^3+^-TPTZ) to ferrous tripyridyltriazine (Fe^2+^-TPTZ) was used for the determination of FRAP. The freshly prepared FRAP solution was mixed with 20 mM ferric chloride (FeCl_3_) (Sigma-Aldrich, USA), 300 mM acetate buffer (Sigma-Aldrich, USA) and 10 mM TPTZ (Sigma-Aldrich, USA) solutions at a ratio of 1:10:1: respectively. In brief, 190 µL of FRAP reagent was mixed with 10 µL of the extract and incubated at 37 °C for 10 min then, absorbance at 593 nm was measured against a blank. L-Ascorbic acid served as a reference standard and the results were also expressed as EC_50_ values [22].

2,2-Azinobis-3-ethylbenzothiazoline-6-sulfonic acid (ABTS) was used for the determination of the free radical-scavenging activity of the extract. ABTS^•+^ solution was prepared by mixing 7 mM ABTS (Sigma-Aldrich, USA) and 2.45 mM potassium persulfate (K_2_S_2_O_8_) (Sigma-Aldrich, USA) at a ratio of 2:3. In brief, 30 µL of various concentrations of the extracts was mixed with 120 µL of distilled water and 30 µL of ethanol and reacted with 3 mL of ABTS^•+^solution. The absorbance was measured at 734 nm with a spectrophotometer (Pharmacia LKB-Biochrom4060). Trolox was used as the reference standard. The radical scavenging activity was also expressed as EC_50_ value [23].

Cyclo-oxygenase-II (COX-II) inhibition was determined using a colorimetric COX-II inhibitor screening assay kit (Cayman Chemical, USA). The antiinflammation effect of the extracts on COX-II inhibition activity was assayed according to the manufacture’s protocol. COX-II working solution was prepared by dissolving COX-II substance in 100 mM Tris–HCl buffer, pH 8.0 at a ratio of 1:100. In brief, the mixed solution containing 150 µL of assay buffer, 10 µL of extract, 10 µL of heme (Cayman Chemical, USA), 10 µL of COX-II working solution, 20 µL of 10 µM TMPD (N,N,N',N'-Tetramethyl-p-phenylenediamine dihydrochloride) (Sigma, USA) and 20 µL of 100 µM arachidonic acid (Cayman Chemical, USA) was added to 96-well microliter plates and incubated at 25 °C for 30 min. Then, absorbance at 590 nm was measured. Indomethacin served as a reference standard. The results were expressed as EC_50_ [24].

### Determination of combination index (CI) and dose reduction index (DRI)

Based on the synergistic effect between mulberry fruit extract and mulberry leaf extract, the combination index (CI) was calculated as follows:

$$\mathrm{CI}=(\mathrm{D}1/\mathrm{Dx}1) + (\mathrm{D}2/\mathrm{Dx}2)$$

Dx_1_ and Dx_2_ were the concentration of mulberry fruit extract and mulberry leaf extract used in combination to achieve half-maximal response. D_1_ and D_2_ were the concentrations of single extract required to achieve the same effect. The CI values of < 1, = 1, and > 1.45 indicated synergism, an additive effect, and antagonism of the interaction, respectively [25].

The dose which might be reduced in combination for a given level of effect compared to the concentration of individual extract alone**,** was defined as dose reduction index (DRI) and calculated as follows:

$$\mathrm{DRI}=\mathrm{Dx}1/\mathrm{D}1$$

Dx_1_ was the concentration of the extract alone used in combination to achieve half-maximal response. D_1_ was the concentration for single extract to achieve the same effect [26].

### Cell culture and cell viability assay

#### Cell culture

The human neuroblastoma SH-SY5Y cell line, derived from a neuroblastoma bone marrow biopsy, is of neuronal origin and exhibits neuronal cell properties. It was obtained from the American Type Culture Center (SH-SY5Y, Cat. No. CRL-2266, ATCC Manassas, Virginia). The SH-SY5Y cell line was maintained in DMEM medium (Gibco, USA) supplemented with 10% FBS, 1% penicillin–streptomycin and 1% non-essential amino acids at 37˚C in a humidified atmosphere containing 5% CO_2_. Cells were plated at an appropriate density according to each experiment. At the beginning of each experiment, the culture medium in each well was completely removed and replaced with fresh medium containing hydrogen peroxide with or without MFML.

#### Cell viability assay

The in vitro cytotoxicity of hydrogen peroxide and MFML were determined using the MTT assay. SH-SY5Y cells were plated in 96-well plates at a density of 1 × 10^4^ cells/well and cultured as described above. Cells were treated with various concentrations of hydrogen peroxide (50–1,000 µM) and MFML (0–1,000 µg/mL) in serum free DMEM for 24 h. To determine whether MFML protects against hydrogen peroxide-induced neurotoxicity, cells were pre-treated with MFML for 6 h. Then, media was removed and fresh medium containing hydrogen peroxide with or without MFML was replaced. Following 24 h of treatment (18 h after incubation with 200 µM of hydrogen peroxide), the medium was removed and replaced with MTT reagent (Sigma, USA) at a final concentration of 0.5 mg/mL. Cells were then incubated at 37˚C for 1 h in a humidified atmosphere containing 5% CO_2_ in an incubator. After incubation, MTT reagent was aspirated and 100 µL of dimethyl sulfoxide was added to dissolve the insoluble purple formazan product. The absorbance at 570 nm was determined.

### Assessment of oxidative stress status

The treated cells were homogenized with 0.1 M potassium phosphate buffer solution, pH 7.4 (sample dilution 10 mg: PBS 50 µL). The protein concentrations in cell homogenates were assessed by using a Thermo Scientific NanoDrop 2000c spectrophotometer (Thermo Fisher Scientific, Wilmington, Delaware, USA), and the optical absorbance measured at 280 nm.

Catalase (CAT) activity was determined based on the ability of the enzyme to break down hydrogen peroxide. In brief, 10 µL of the prepared sample was mixed with a solution containing 25 µL of 4 M H_2_SO_4_ (Sigma-Aldrich, USA), 50 µL of 30 mM hydrogen peroxide (in 50 mM phosphate buffer, pH7.0) (BDH Chemicals Ltd, UK) and 150 µL of 5 mM KMnO_4_ (Sigma-Aldrich, USA). Following this step, the absorbance at 490 nm was measured. CAT enzyme (Sigma-Aldrich, USA) was used as a reference standard at concentrations ranging from 10–100 units/mL. The result was expressed as units/mg protein [27].

The method of Sun and coworkers was used for the determination of superoxide dismutase (SOD) activity. In brief, 0.5 mM of xanthine, pH 7.4 (Sigma-Aldrich, USA), 0.2 M phosphate buffer solution (KH_2_PO_4_), pH 7.8 (Sigma-Aldrich, USA), 0.01 M EDTA (Sigma-Aldrich, USA) and 15 M cytochrome C (Sigma-Aldrich, USA) were mixed at the ratio of 50:25:1:1 (v/v). The prepared sample (20 µL) was mixed with 200 µL of the mixture solution and 20 µL of xanthine oxidase (0.90 mU/mL) (Sigma-Aldrich, USA) and absorbance was determined at 415 nm. SOD enzyme (Sigma-Aldrich, USA) activity at concentrations ranging from 0–25 units/mL were used as the reference standard. The results were also expressed as units/mg protein [28].

The glutathione peroxidase (GSH-Px) activity was assessed by mixing 20 µL of the prepared sample with the mixed solution containing 10 µL of 1 mM dithiothreitol (DTT) (Sigma-Aldrich, USA) and 10 mM monosodium phosphate (NaH_2_PO_4_) in DW, 1 mM sodium azide (Sigma-Aldrich, USA) 100 µL in 40 mM potassium phosphate buffer (pH 7.0), 10 µL of 50 mM glutathione (Sigma-Aldrich, USA) solution and 100 µL of 30% hydrogen peroxide (BDH Chemicals Ltd, UK). The reaction mixture was incubated at 25 °C for 10 min. Then, 10 µL of 10 mM DTNB (5,5-dithiobis-2-nitrobenzoic acid) (Sigma-Aldrich, USA) was added. The absorbance at 412 nm was measured. GSH-Px enzyme (Sigma-Aldrich, USA) activity at concentrations ranging from 1–5 units/mL was used as a reference standard. The GSH-Px activity was expressed as units/mg protein [29].

Malondialdehyde (MDA), a marker of lipid peroxidation, was measured using a thiobarbituric acid reaction. In brief, 50 µL of the prepared sample was mixed with a mixed solution containing 50 µL of 8.1% sodium dodecyl sulfate (Sigma-Aldrich, USA), 375 µL of 0.8% thiobarbituric acid (Sigma-Aldrich, USA), 375 µL of 20% acetic acid (Sigma-Aldrich, USA) and 150 µL of distilled water, then maintained at 95 °C for 60 min. Following this, the reaction mixture was cooled with tap water and mixed with the solution containing 1,250 µL of n-butanol and pyridine (Merck, Germany) and 250 µL of DW at a ratio of 15: 1. Then, the mixed solution was subjected to 4,000-rpm centrifugation for 10 min. The upper layer was harvested and the absorbance at 532 nm was measured. TMP (1,1,3,3-tetra methoxy propane) (0–15 µM) (Sigma-Aldrich, USA) was used as reference standard. MDA was expressed as ng/mg protein [30].

### Western blotting analysis

The treated cell was lysed in 1/5 (w/v) RIPA (radioimmunoprecipitation assay) buffer (Cell Signaling Technology, USA) containing 20 mM Tris–HCl (pH 7.5), 150 mM NaCl, 1 mM Na_2_EDTA, 1 mM EGTA, 1% NP-40, 1% sodium deoxycholate, 2.5 mM sodium pyrophosphate, 1 mM beta-glycerophosphate, 1 mM Na_3_VO_4_, 1 µg/ml leupeptin and 1 mM phenylmethanesulfonyl fluoride (PMSF) (Cell Signaling Technology, USA). The prepared sample was centrifuged at 12,000 X g- at 4 °C for 10 min and the supernatant retained. Protein concentration was determined using a Thermo Scientific NanoDrop 2000c spectrophotometer (Thermo Fisher Scientific, Wilmington, Delaware, USA). 80 μg of cell lysate was adjusted to the appropriate concentration by using Tris–Glycine SDS-PAGE loading buffer and heated at 95 °C for 10 min. Protein in the cell lysate was isolated using sodium dodecyl sulfate–polyacrylamide gel electrophoresis (SDS-PAGE) by loading 20 µL of cell lysate on SDS–polyacrylamide gel. Then, the separated bands were transferred to a nitrocellulose membrane, washed with 0.05% TBS-T, and incubated in blocking buffer (5% skim milk in 0.1% TBS-T) at 25 °C for 1 h. After this step, the nitrocellulose membrane was incubated with anti-NF-κB (Cell Signaling Technology, USA; dilution 1:500), Anti-TNF-α (Cell Signaling Technology, UK; dilution 1:500), Anti-BCL2 (Cell Signaling Technology, USA; dilution 1:500), Anti-Caspase-3 (Cell Signaling Technology, USA; dilution 1:500), Anti-Caspase-9 (Cell Signaling Technology, USA; dilution 1:500) and anti-β-actin (Cell Signaling Technology, USA; dilution 1:1000) antibodies at 4 °C, overnight. After incubation, the nitrocellulose membrane was again rinsed with TBS-T (0.05%) and incubated with anti-rabbit IgG, HRP-linked antibody (Cell Signaling Technology, USA; dilution 1:2000) at 25 °C for 1 h. The bands were visualized and quantitated by using the ECL detection system (GE Healthcare) and LAS-4000 luminescent image analyzer (GE Healthcare). Band intensities were measured for statistical analysis using ImageQuant TL v.7.0 image analysis software (GE Healthcare). The expression was normalized using anti-β-actin. The membranes were incubated and visualized with each antibody followed by the internal control. Representative Western blot images were cropped in order to remove irrelevant parts of the captured image and present only the proteins of interest, and full-length blots are presented in supplementary material. Data were presented as a relative density to the naïve control group [31].

### Statistical analysis

All data were expressed as mean ± standard error of mean (SEM). Statistical significance was evaluated by using one-way analysis of variance (ANOVA), followed by the post hoc (Tukey) test. The Student's t test was used for comparison between the means for the two groups. The statistical significance set at *p*-values < 0.05. All statistical data analyses were performed using SPSS version 21.0 (IBM Corp. Released 2012. IBM SPSS Statistics for Windows) [8, 12, 31].

## Results

### Determination of active compounds and biological activities

Table 1 shows the contents of the important active ingredients in mulberry fruit extract, mulberry leaf extract, and the combined extract of mulberry fruit and mulberry leaf (MFML). It was found that mulberry fruit extract contained total phenolic, flavonoid, and anthocyanins content at the concentrations of 217.00 ± 3.33 mg GAE/mg extract, 159.33 ± 4.01 mg quercetin/mg extract and 270.33 ± 4.19 mg C3G /mg extract, respectively. Content of total phenolics, flavonoid, and anthocyanins in mulberry leaf extract was 200.33 ± 3.85 mg GAE/mg extract, 128.22 ± 1.70 mg quercetin/mg extract and 25.33 ± 0.96 mg C3G /mg extract, respectively. Interestingly, the content of flavonoids and anthocyanins in MFML was significantly higher than that in mulberry leaf extract (*p* < 0.01 and 0.001, respectively) but no significant difference was observed in all substances just mentioned between mulberry fruit extract and MFML.

**Table 1:** Phenolic compositions and biological activities of mulberry fruit extract, mulberry leaf extract and the combined extract of mulberry fruit and mulberry leaf

Parameters	Units	Mulberry fruit extract	Mulberry leaf extract	The combined extract of mulberry fruit and mulberry leaf	Standard reference
**Active compounds**					
Total phenolic content	mg Gallic acid/mg	217.00 ± 3.33	200.33 ± 3.85	219.83 ± 0.05	-
Total flavonoid content	mg Quercetin/mg	159.33 ± 4.01	128.22 ± 1.70	161.11 ± 2.96^**^	-
Total anthocyanins content	mg C3G /mg	270.33 ± 4.19	25.33 ± 0.96	271.83 ± 0.42^***^	-
**Antioxidant activities**					
DPPH	EC_50_ (mg/mL)	151.25 ± 3.36	187.86 ± 3.32	72.73 ± 3.56^aaa, ***^	12.45 ± 0.01, Trolox
FRAP	EC_50_ (mg/mL)	72.85 ± 0.52	144.90 ± 1.84	63.88 ± 1.27^***^	Trolox
ABTS	EC_50_ (mg/mL)	106.07 ± 2.97	184.47 ± 1.49	77.18 ± 0.82^aaa, ***^	33.67 ± 0.02, Trolox
**Inflammatory marker**					
COX-II	EC_50_ (mg/mL)	51.55 ± 0.16	52.62 ± 0.36	48.33 ± 0.20	12.22 ± 0.01, Indomethacin

Data are presented as mean ± SEM. ^aaa^*p* < 0.001; compared between compared between mulberry fruit extract and the combined extract of mulberry fruit and mulberry leaf, ^**^, ^***^p < 0.01 and 0.001, respectively; compared between mulberry leaf extract and the combined extract of mulberry fruit and mulberry leaf

The combined extract of mulberry fruit and mulberry leaf was developed based on the synergistic concept. To determine the synergistic effect, the biological activities associated with the pathophysiology of dementia including antioxidant and suppression effects of COX-2 were explored and the results are shown in Table 1. The data showed that EC_50_ of the antioxidant activities via DPPH and ABTS assay of MFML was significantly higher than both mulberry fruit extract and mulberry leaf extract (*p* < 0.001 all). Significant differences of antioxidant activity via FRAP assay between MFML and mulberry leaf extract were also observed (*p* < 0.001) but no significant difference of EC_50_ of the antioxidant activity via FRAP assay between MFML and mulberry fruit extract was observed. The potent suppression activity of COX-2 was also determined. The data showed that MFML was the most potent extract. However, no significant differences were observed.

To confirm the synergistic effect of MFML, the combination indices were also explored and used as indicators. The results are shown in Table 2. Regarding antioxidant effect, the combination indices of MFML *via* DPPH, FRAP, and ABTS assays were 0.87 ± 0.02, 1.32 ± 0.02 and 0.99 ± 0.0, respectively. In addition, the combination index of the suppression effects on COX-2 of MFML was 1.86 ± 0.00. To assure that the interaction between ingredients of MFML can provide beneficial effects, the dose reduction indices of the biological activities were also monitored. For antioxidant activities *via* DPPH, FRAP, and ABTS assays, dose reduction indices of mulberry fruit extract in MFML were 2.09 ± 0.04, 1.14 ± 0.02 and 1.37 ± 0.01whereas the afore mentioned parameters of mulberry leaf extract in MFML were 2.59 ± 0.05, 2.27 ± 0.04 and 2.39 ± 0.01. The dose reduction index of mulberry fruit extract in MFML concerning COX-2 suppression activity was 1.07 ± 0.00, whereas the dose reduction index of mulberry leaf extract in MFML for this activity was 1.09 ± 0.00. The current data demonstrated that combination indices of MFML *via* DPPH, FRAP, and ABTS assays were in the range between 0.87- 1.32. These indicate synergistic effects of the various components in MFML. The combination index of the suppression effects on COX-2 of MFML was more than 1.45. This indicates antagonism. In addition, dose reduction indices of both extracts in MFML were more than 1. These also indicate the reduction of the effective dose.

**Table 2:** The combination index (CI) values of the combined extract of mulberry fruit and mulberry leaf and dose reduction index (DRI) value of mulberry fruit extract and mulberry leaf extract

**Parameters** EC_50_ (mg/ml)	**Mulberry fruit extract**	**Mulberry leaf extract**	**The combined extract of mulberry fruit and mulberry leaf**	**Combination index** **(Type of interaction)**	**Dose reduction index**	
					**Mulberry fruit extract**	**Mulberry leaf extract**
DPPH	151.25 ± 3.36	187.86 ± 3.32	72.73 ± 3.56	0.87 ± 0.02 (synergism)	2.09 ± 0.04	2.59 ± 0.05
FRAP	72.85 ± 0.52	144.90 ± 1.84	63.88 ± 1.27	1.32 ± 0.02 (additive)	1.14 ± 0.02	2.27 ± 0.04
ABTS	106.07 ± 2.97	184.47 ± 1.49	77.18 ± 0.82	0.99 ± 0.01 (synergism)	1.37 ± 0.01	2.39 ± 0.01
COX-II	51.55 ± 0.16	52.62 ± 0.36	48.33 ± 0.20	1.86 ± 0.00 (antagonism)	1.07 ± 0.00	1.09 ± 0.00

Data are presented as mean ± SEM

### Cytotoxicity of hydrogen peroxide and MFML on the viability of SH‐SY5Y cells

**Fig. 1:**
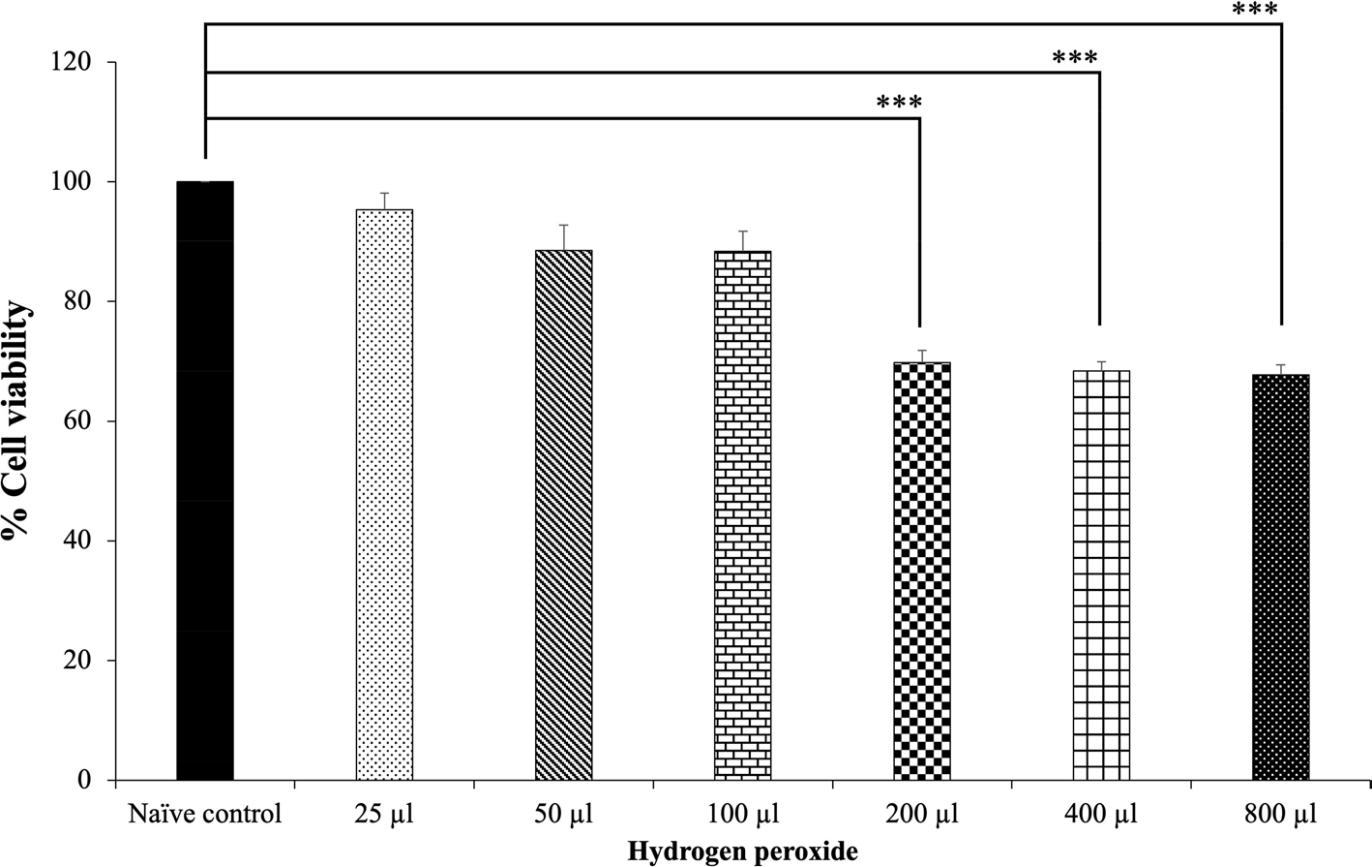
Effect of hydrogen peroxide on the viability of SH-SY5Y cells. Data are presented as mean ± SEM. ^***^p < 0.001; compared with naïve control

It has been reported that hydrogen peroxide treatment decreased the viability of SH-SY5Y cells, induced apoptosis, decreased scavenging enzyme activity [32], and induced inflammation [33]. Therefore, the hydrogen peroxide-induced SH-SY5Ycytotoxixity model was used for the determination of the cytoprotective effect of MFML. To determine the optimal concentration of hydrogen peroxide treatment to elicit toxic effects on SH-SY5Y cells, cells were treated with 25, 50, 100, 200, 400, and 800 µM hydrogen peroxide for 24 h. Cell viability was measured using the MTT assay and the results are shown in Fig. 1 revealing that cell viability was 95.37 ± 2.76, 88.55 ± 4.24, 88.35 ± 3.39, 69.77 ± 2.07, 68.37 ± 1.58 and 67.74 ± 1.72, respectively. A significant decrease of cell viability was observed in hydrogen peroxide treatment at doses of 200, 400, and 800 µM (*p* < 0.001 all; compared to the naïve control group). Based on these data, treatment with 200 µM hydrogen peroxide for 24 h was considered the optimal concentration which was employed in subsequent experiments.

**Fig. 2:**
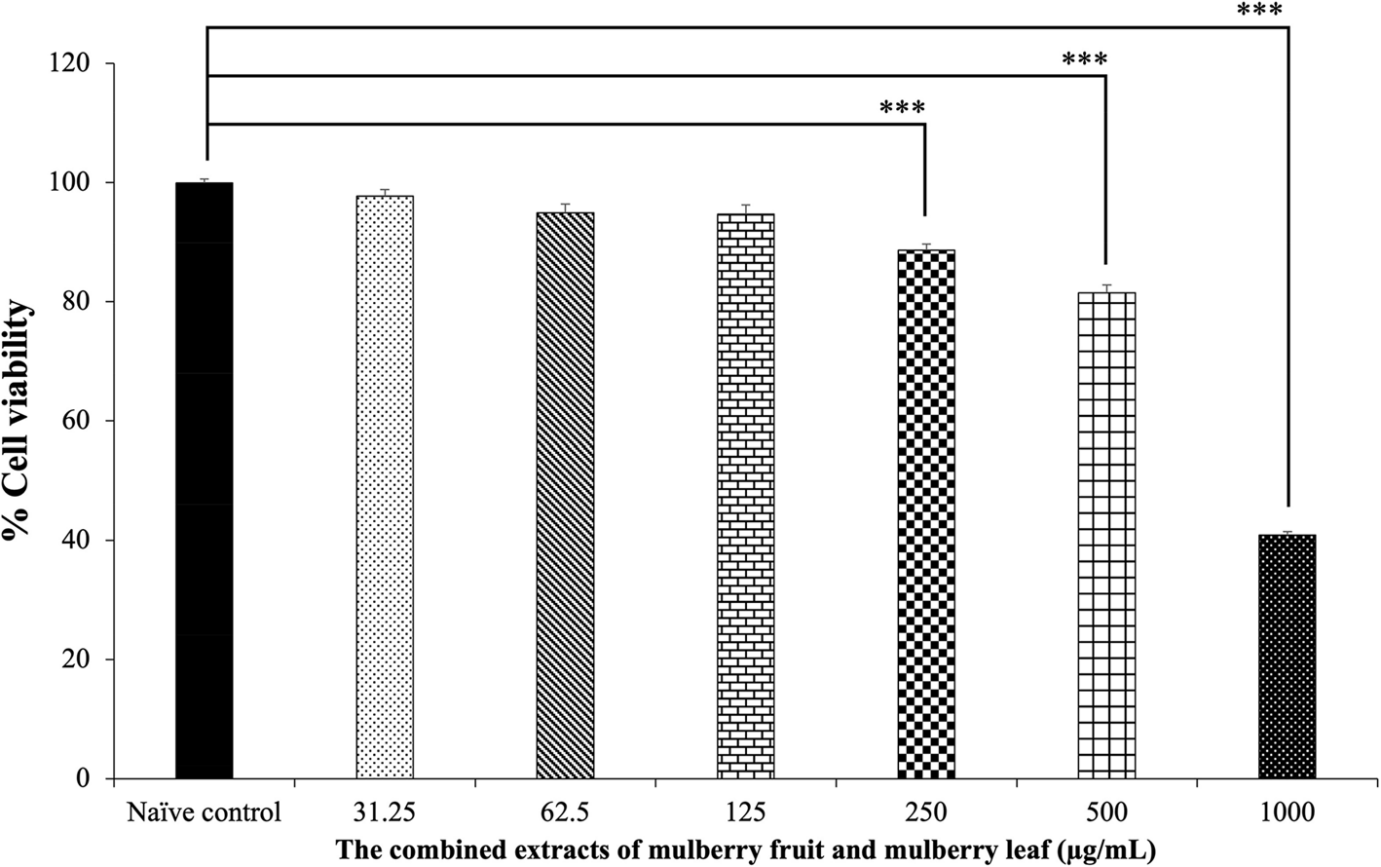
Effect of the combined extract of mulberry fruit and mulberry leaf on the viability of SH-SY5Y cells. Data are presented as mean ± SEM. ^***^p < 0.001; compared with naïve control

To assure the cytotoxicity of MFML, SH-SY5Ycells were treated with MFML at doses of 31.25, 62.5, 125, 250, 500, and 1,000 μg/mL for 24 h, and the results are shown in Fig. 2. It was found that cell viability was 97.71 ± 1.22, 94.96 ± 1.45, 94.70 ± 1.53, 88.63 ± 1.06, 81.48 ± 1.37 and 40.91 ± 0.57, respectively. A significant reduction of cell viability was observed in MFML treatment at doses of 250, 500, and 1,000 μg/mL (*p* < 0.001 all; compared to the naïve control group). Therefore, MFML treatments at doses of 62.5 and 125 μg/mL were the maximum doses which were non-toxic to SH-SY5Y cells and were selected for determining the protective effect against hydrogen peroxide-induced cytotoxicity.

### Effects of MFML on the viability of SH‐SY5Y cells

**Fig. 3:**
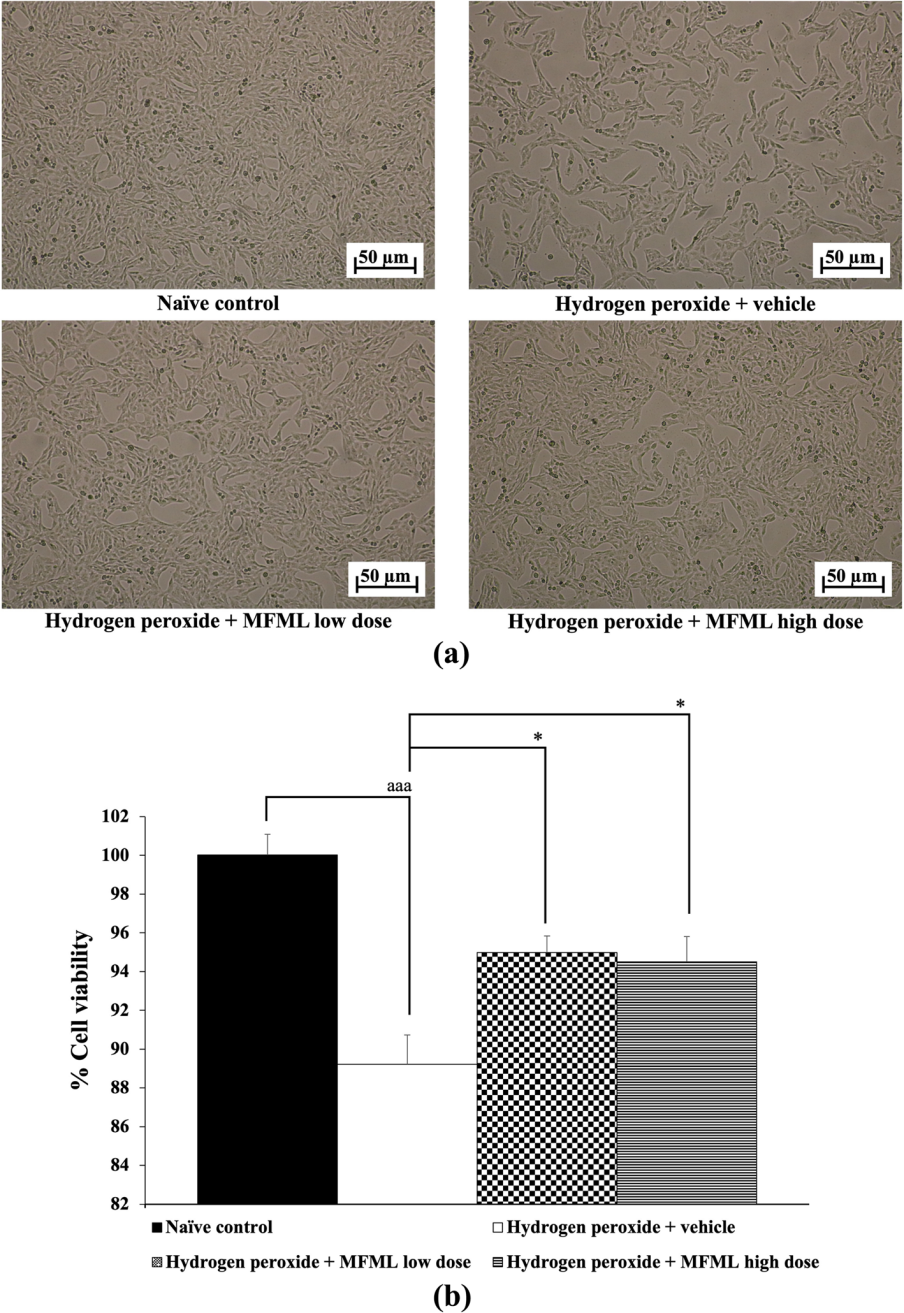
Effect of MFML on hydrogen peroxide induced cytotoxicity in SH-SY5Y cells. (**a**) Light microscopy of SH-SY5Y cell morphology at 40 × magnification. (**b**) % cell viability of SH-SY5Y cells. Data are presented as mean ± SEM. ^aaa^*p* < 0.001; compared to naïve control, ^*^p < 0.05; compared to SH-SY5Y cells treated with hydrogen peroxide, and which had received the vehicle. hydrogen peroxide: hydrogen peroxide at dose of 200 μg/mL; MFML low dose and MFMF high dose: the combined extract of mulberry fruit and mulberry leaf at doses of 62.5 and 125 μg/mL, respectively

The protective effect of MFML is shown in Fig. 3. The results revealed that SH-SY5Y cells treated with hydrogen peroxide and which had received the vehicle had significantly decreased cell viability (*p* < 0.001 compared to the naïve control group). Interestingly, this decrease was significantly reversed by all doses of MFML treatment (*p* < 0.05 all; compared to hydrogen peroxide + vehicle group).

### Effects of MFML on the oxidative stress status

Table 3 shows the effects of MFML on oxidative stress status, including the level of MDA and the activities of the main scavenger enzymes (CAT, SOD, and GSH-Px). It was found that SH-SY5Y cells treated with hydrogen peroxide and had received the vehicle had significantly increased MDA level (*p* < 0.001; compared to naïve control group) but decreased CAT, SOD and GSH-Px activities (*p* < 0.05, 0.01 and 0.01, respectively; compared to naïve control group). SH-SY5Y cells treated with hydrogen peroxide which had received MFML at dose of 62.5 μg/mL showed significantly increased SOD and GSH-Px activities but decreased MDA level (*p* < 0.05, 0.05 and 0.01, respectively; compared to hydrogen peroxide + vehicle group). MFML treatment at doses of 125 μg/mL also produced a significant decrease in MDA level (*p* < 0.05; compared to hydrogen peroxide + vehicle group) together with the significant increase in GSH-Px activity (*p* < 0.05; compared to hydrogen peroxide + vehicle group). However, no significant change of CAT activity was observed in SH-SY5Y cells treated with hydrogen peroxide and those that received MFML.

**Table 3:** The effect of the combined extract of mulberry fruit and mulberry leaf on oxidative stress markers in SH-SY5Y cell toxicity induced by hydrogen peroxide

Treatment groups	MDA (ng/mg protein)	CAT (units/mg protein)	SOD (units/mg protein)	GSH-Px (units/mg protein)
Naïve control	16.98 ± 0.96	44.08 ± 1.04	18.03 ± 1.74	24.76 ± 1.60
Hydrogen peroxide + vehicle	37.66 ± 4.35^aaa^	27.38 ± 4.03^a^	11.24 ± 0.84^aa^	15.87 ± 1.79^aa^
Hydrogen peroxide + MFML low dose	22.57 ± 2.21^**^	36.74 ± 4.43	16.89 ± 1.34^*^	22.38 ± 0.95^*^
Hydrogen peroxide + MFML high dose	26.01 ± 1.56^*^	37.20 ± 7.98	12.32 ± 0.85^a^	22.21 ± 1.09^*^

Data are presented as mean ± SEM. ^a, aa, aaa^*p* < 0.05, 0.01 and 0.001, respectively; compared to naïve control, ^*, **^p < 0.05 and 0.01, respectively; compared to SH-SY5Y cells treated with hydrogen peroxide, and which had received the vehicle. MFML low dose and MFML high dose: the combined extract of mulberry fruit and mulberry leaf at doses of 62.5 and 125 μg/mL, respectively

### Effects of MFML on inflammatory markers

**Fig. 4:**
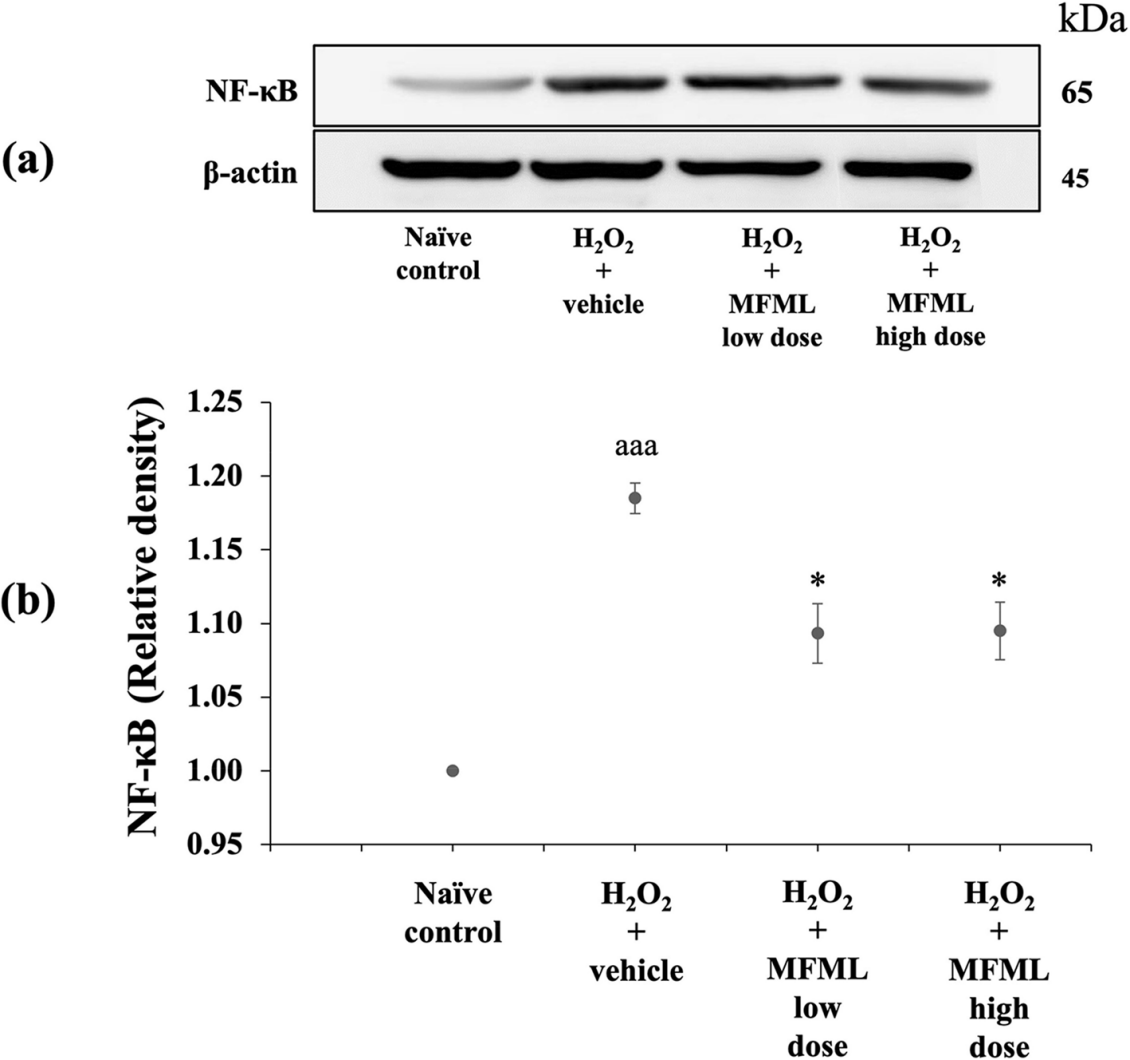
Effect of MFML on the expression of NF-κB in the hydrogen peroxide induced SH-SY5Y cell toxicity was detected by Western blotting. (**a**) Representative Western blot showing the levels of total NF-κB. (**b**) Relative density of total NF-κB. Data are presented as mean ± SEM. ^aaa^*p* < 0.001; compared to naïve control, ^*^p < 0.05; compared to SH-SY5Y cells treated with hydrogen peroxide, and which had received the vehicle. H_2_O_2_: hydrogen peroxide at dose of 200 μg/mL; MFML low dose and MFMF high dose: the combined extract of mulberry fruit and mulberry leaf at doses of 62.5 and 125 μg/mL, respectively

**Fig. 5:**
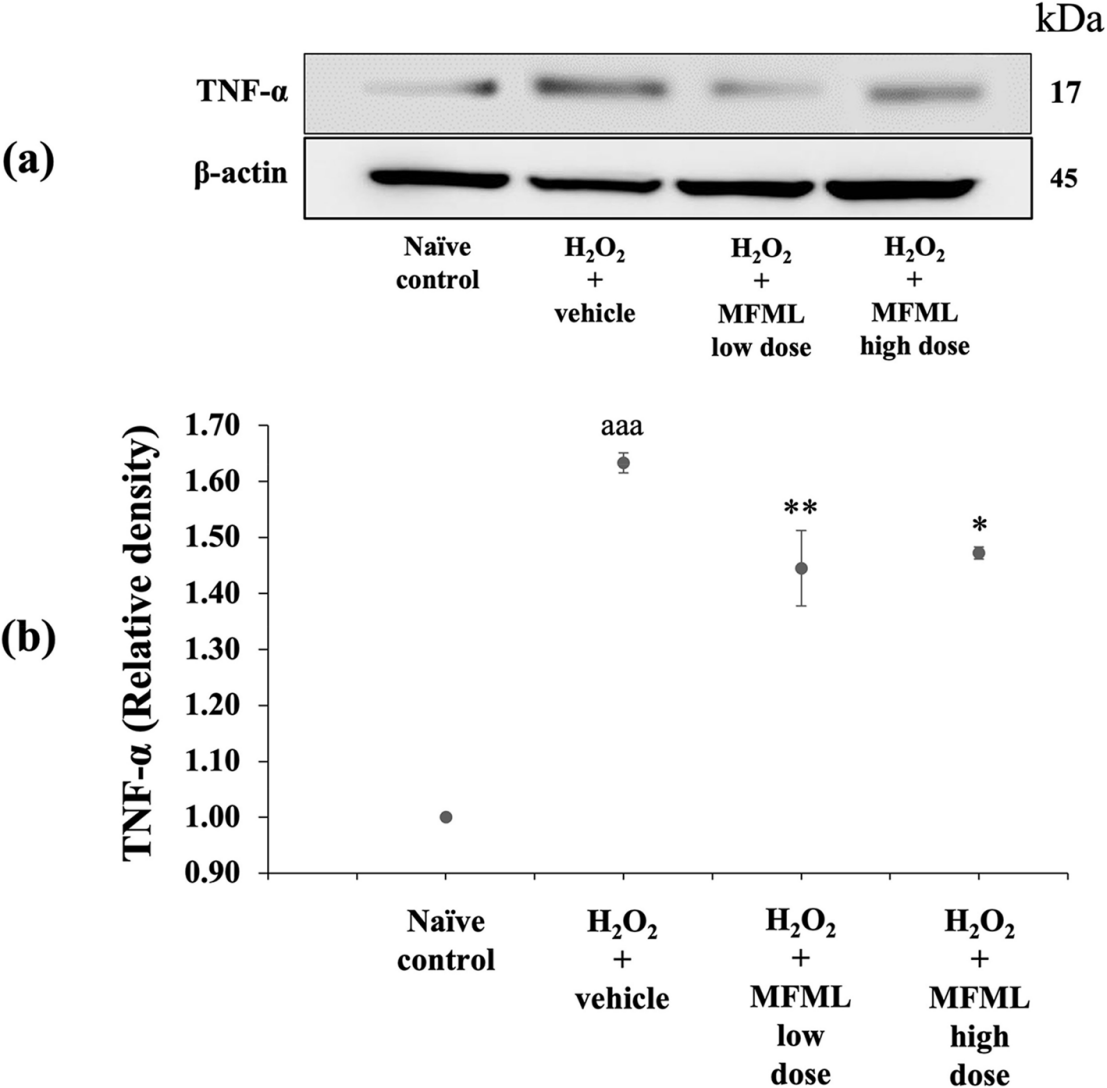
Effect of MFML on the expression of TNF-α in the hydrogen peroxide induced SH-SY5Y cell toxicity was detected by Western blotting. (**a**) Representative Western blot showing the levels of total TNF-α. (**b**) Relative density of total TNF-α. Data are presented as mean ± SEM. ^aaa^*p* < 0.001; compared to naïve control, ^*, **^p < 0.05 and 0.01, respectively; compared to SH-SY5Y cells treated with hydrogen peroxide, and which had received the vehicle. H_2_O_2_: hydrogen peroxide at dose of 200 μg/mL; MFML low dose and MFMF high dose: the combined extract of mulberry fruit and mulberry leaf at doses of 62.5 and 125 μg/mL, respectively

Since neuroinflammation plays a crucial role on the pathophysiology of dementia [34], we also investigated the effect of MFML on inflammatory markers such as NF-κB and TNF-α in the hydrogen peroxide induced SH-SY5Y cytotoxicity, and the results are shown in Figs. 4 and 5. It was found that SH-SY5Y cells treated with hydrogen peroxide and which had received the vehicle significantly enhanced both NF-κB and TNF-α expressions (*p* < 0.001 all; compared to naïve control group). Interestingly, this elevation of NF-κB expression was significantly attenuated by all doses of MFML treatment (*p* < 0.05 all; compared to hydrogen peroxide + vehicle group). In addition, the elevation of TNF-α expression was also significantly counteracted by all doses of MFML treatment (*p* < 0.01 and 0.05, respectively; compared to hydrogen peroxide + vehicle group).

### Effects of MFML on apoptotic markers

**Fig. 6:**
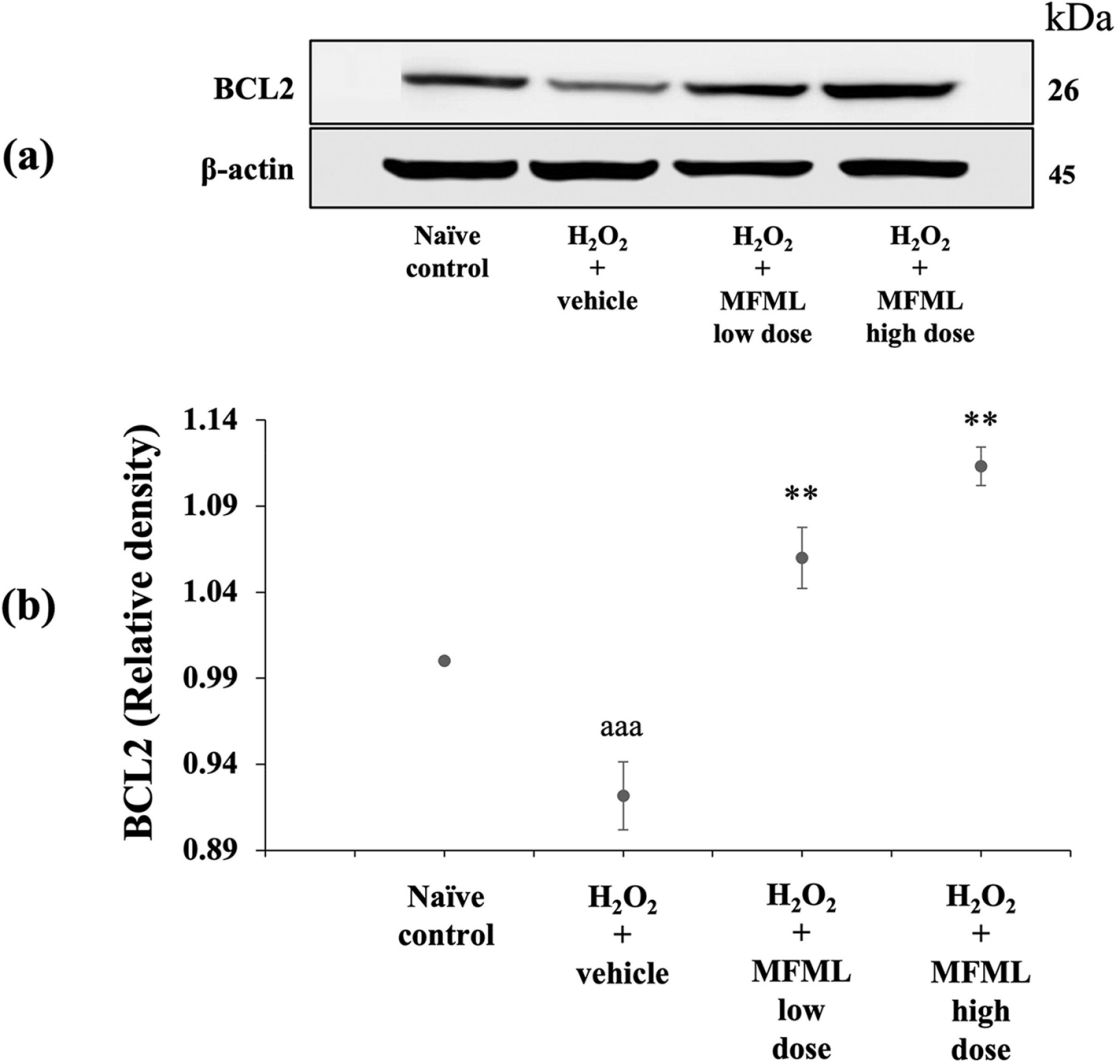
Effect of MFML on the expression of BCL2 in the hydrogen peroxide induced SH-SY5Y cell toxicity was detected by Western blotting. (**a**) Representative Western blot showing the levels of total BCL2. (**b**) Relative density of total BCL2. Data are presented as mean ± SEM. ^aaa^*p* < 0.001; compared to naïve control, ^**^p < 0.01; compared to SH-SY5Y cells treated with hydrogen peroxide, and which had received the vehicle. H_2_O_2_: hydrogen peroxide at dose of 200 μg/mL; MFML low dose and MFMF high dose: the combined extract of mulberry fruit and mulberry leaf at doses of 62.5 and 125 μg/mL, respectively

**Fig. 7:**
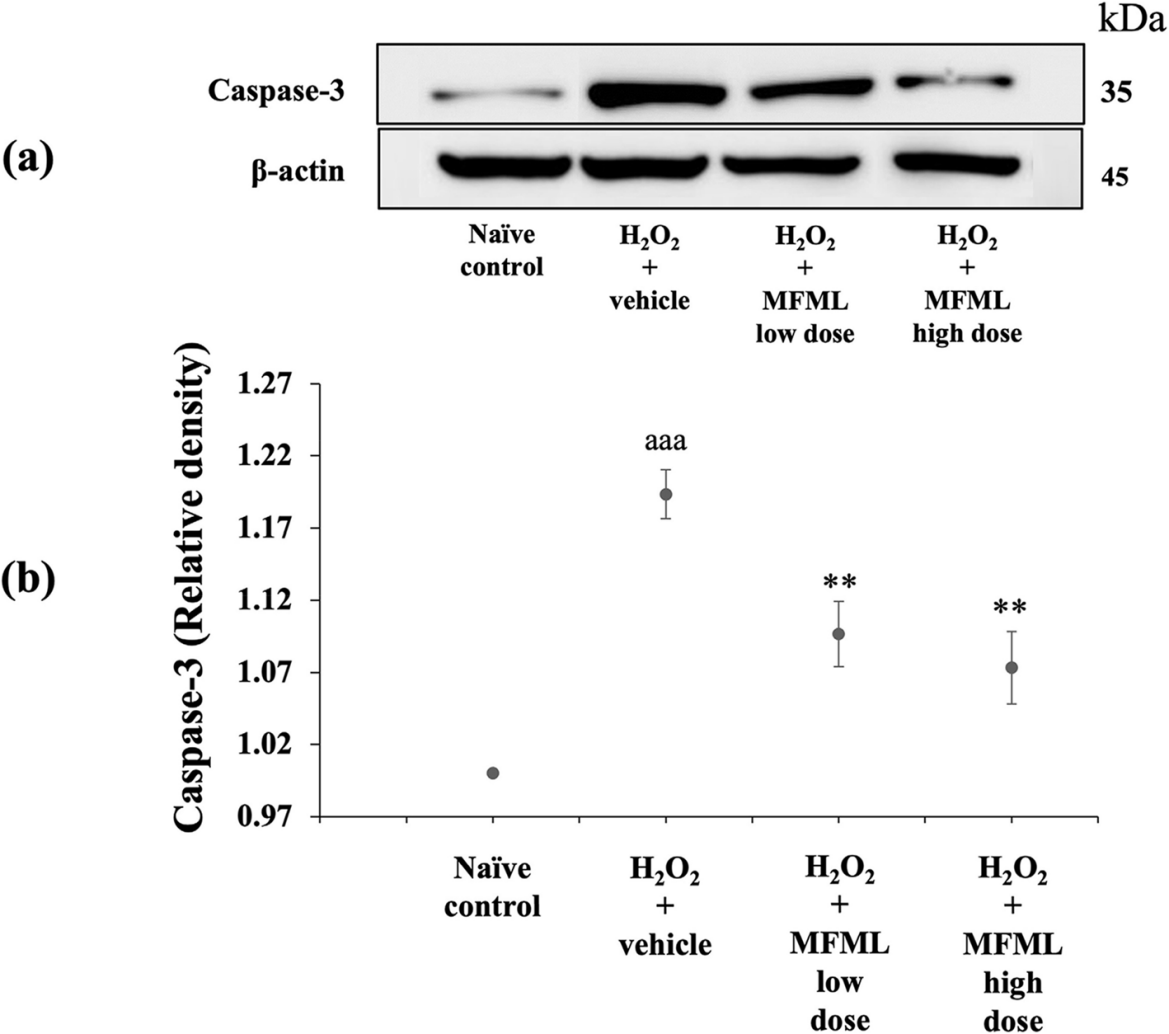
Effect of MFML on the expression of caspase-3 in the hydrogen peroxide induced SH-SY5Y cell toxicity was detected by Western blotting. (**a**) Representative Western blot showing the levels of total caspase-3. (**b**) Relative density of total caspase-3. Data are presented as mean ± SEM. ^aaa^*p* < 0.001; compared to naïve control, ^**^ < 0.01; compared to SH-SY5Y cells treated with hydrogen peroxide, and which had received the vehicle. H_2_O_2_: hydrogen peroxide at dose of 200 μg/mL; MFML low dose and MFMF high dose: the combined extract of mulberry fruit and mulberry leaf at doses of 62.5 and 125 μg/mL, respectively

**Fig. 8:**
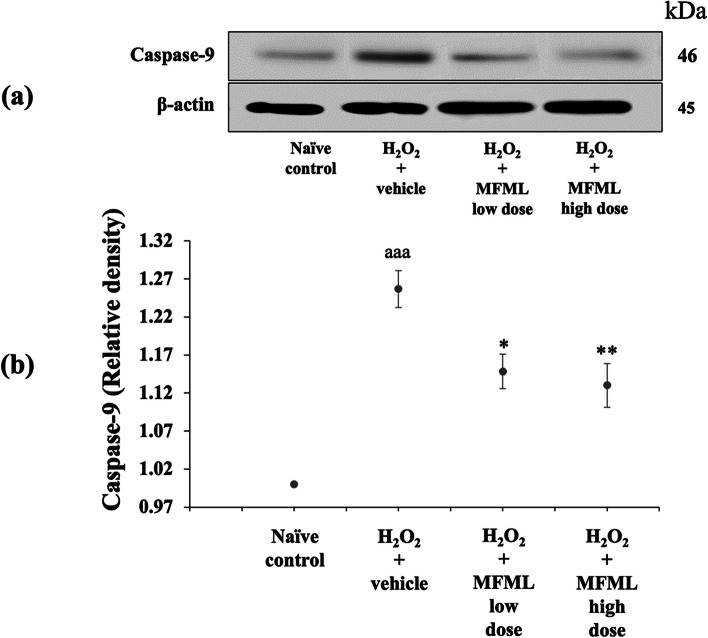
Effect of MFML on the expression of caspase-9 in the hydrogen peroxide induced SH-SY5Y cell toxicity was detected by Western blotting. (**a**) Representative Western blot showing the levels of total caspase-9. (**b**) Relative density of total caspase-9. Data are presented as mean ± SEM. ^aaa^*p* < 0.001; compared to naïve control, ^*, **^p < 0.05 and 0.01, respectively; compared to SH-SY5Y cells treated with hydrogen peroxide, and which had received the vehicle. H_2_O_2_: hydrogen peroxide at dose of 200 μg/mL; MFML low dose and MFMF high dose: the combined extract of mulberry fruit and mulberry leaf at doses of 62.5 and 125 μg/mL, respectively

It has been reported that undesired apoptosis is a factor in many conditions including neurodegenerative diseases [35]. To explore the neuroprotective mechanism of MFML, the apoptotic markers BCL2, caspase-3, and caspase-9 were determined. The results shown in Figs. 6, 7 and 8 indicated that SH-SY5Y cells treated with hydrogen peroxide and which had received the vehicle had significantly decreased BCL2 expression but increased caspase-3 and caspase-9 expression (*p* < 0.001 all; compared to naïve control group). However, this decrease of BCL2 expression was counteracted by all doses of MFML treatment (*p* < 0.01 all; compared to hydrogen peroxide + vehicle group). In addition, all doses of MFML treatment significantly suppressed the increase of caspase-3 expression (*p* < 0.01 all; compared to hydrogen peroxide + vehicle group) and caspase-9 expression (*p* < 0.05 and 0.01, respectively; compared to hydrogen peroxide + vehicle group).

## Discussion

Polyherbal formulations, which contains two or more medicinal plant extracts, have long-been used for treating numerous diseases in Asian countries. Our data showed that MFML contained more polyphenolic compounds, flavonoids, and anthocyanins compared with single extracts from leaf or fruit. In addition, the data also showed that the EC_50_ of the biological activities associated with the pathophysiology of dementia, including antioxidant and suppression effects of COX-2, were significantly higher than both mulberry fruit extract and mulberry leaf extract. Interestingly, the current data have clearly shown that the combination index (CI) of MFML *via* DPPH and ABTS assay is less than one (< 1) and *via* FRAR < 1.45. These indicate synergistic effects of the various components in MFML. Although, CI value of COX-2 suppression of MFML was more than > 1.45 (antagonism), the EC_50_ of COX-2 suppression of MFML was the most potent extract when compared to individual mulberry fruit extract or mulberry leaf extract. This indicates the combined effect of two extracts is more potent than the individual effects. In addition, it was shown that the dose reduction ratio of MFML is more than one (> 1). These data indicate that the interaction between various components of MFML is a synergistic effect and can reduce the effective dose. Therefore, our data also confirm the increasing benefit of synergistic effects induced by drug-drug interactions [36–38]. The possible explanation for the synergistic effect of mulberry fruit and mulberry leaf extract may be partly associated with the modifying effect of the ingredients on the same targets such as antioxidant and anti-inflammation effects or modulation of pharmacodynamics.

The current results have demonstrated the cytoprotective effect of MFML in hydrogen peroxide induced SH-SY5Y cytotoxicity. In addition, the reduction of MDA level, NF-κB, TNF-α, Casapase-3 and Caspase-9, and the increase in SOD, CAT, and GSH- Px activities, and BCL2 expression were also observed.

Hydrogen peroxide is widely known as one of the most important toxic inducers of oxidative stress, which produces highly reactive hydroxyl radicals, resulting in cellular damage [39]. Moreover, accumulation of hydrogen peroxide has been observed in neurodegenerative diseases with apoptosis and inflammation in the brain [40]. Our results have revealed that SH-SY5Y cells exposed to hydrogen peroxide showed elevated oxidative stress, inflammatory markers, and apoptotic factors. These findings correspond with previous findings reported in the literature [32–34].

It has been reported that hydrogen peroxide induces oxidative damage in neuronal cells *via* overproduction of reactive oxygen species and free radicals, and attenuation of the activities of scavenging enzymes [41]. Here, our results confirmed that hydrogen peroxide treatment reduced CAT, SOD, and GSH-Px activities, and increased MDA level. However, the decrease of SOD and GSH-Px activities and the increase of MDA level were effectively reversed by MFML treatment, suggesting that MFML may increase the activities of scavenger enzymes mentioned earlier, resulting in the reduction of the level of MDA. The reduction of oxidative stress manifested by the decrease in MDA contributed to the important roles on the improvement of neuronal cell injury resulting in increased neural cell survival.

Inflammation of the neuron is also a major cause of neurodegeneration. The overproduction of inflammatory mediators has been shown to be related to neuronal cell injury, memory, and cognitive function decline in dementia [42]. Hydrogen peroxide exposure was shown to induce inflammation *via* excessive production of transcriptional factor (NF-κB) and inflammatory mediator (TNF-α) [43]. In this study, we suggested that the protective effect of MFML is associated with inflammatory suppression manifested by the decrease in NF-κB and TNF-α which in turn decrease cell damage resulting in the improvement of cell viability.

Our results also reveal that hydrogen peroxide treatment can successfully induce undesired apoptosis. These findings correspond with a previous study [44]. In addition, it has been revealed that undesired apoptotic mechanism is under the influence of oxidative stress and inflammation [41]. These changes give rise to the determination of apoptosis, which in turn induces cell death. We have found that hydrogen peroxide exposure induced cell apoptosis through increasing of caspase-3 and caspase-9 levels and decreasing the level of BCL2. Based on this information, we suggest that the positive modulation effect of MFML on the improvement of SH-SY5Y cell viability in this study may have occurred partly *via* the improvement of oxidative stress and inflammation giving rise to downregulation of the expression of caspase-3 and caspase-9, and upregulation of BCL2 which in turn suppress the undesired apoptosis resulting in increased of cell viability.

Taken all together, this study clearly reveals the neuroprotective effect of MFML in hydrogen peroxide induced SH-SY5Y cytotoxicity. The possible underlying mechanism may occur partly *via *the improvement of undesired apoptotic processes and *via* the improvement of oxidative stress status and inflammation leading to the improvement of cell viability.

## Conclusion

**Fig. 9:**
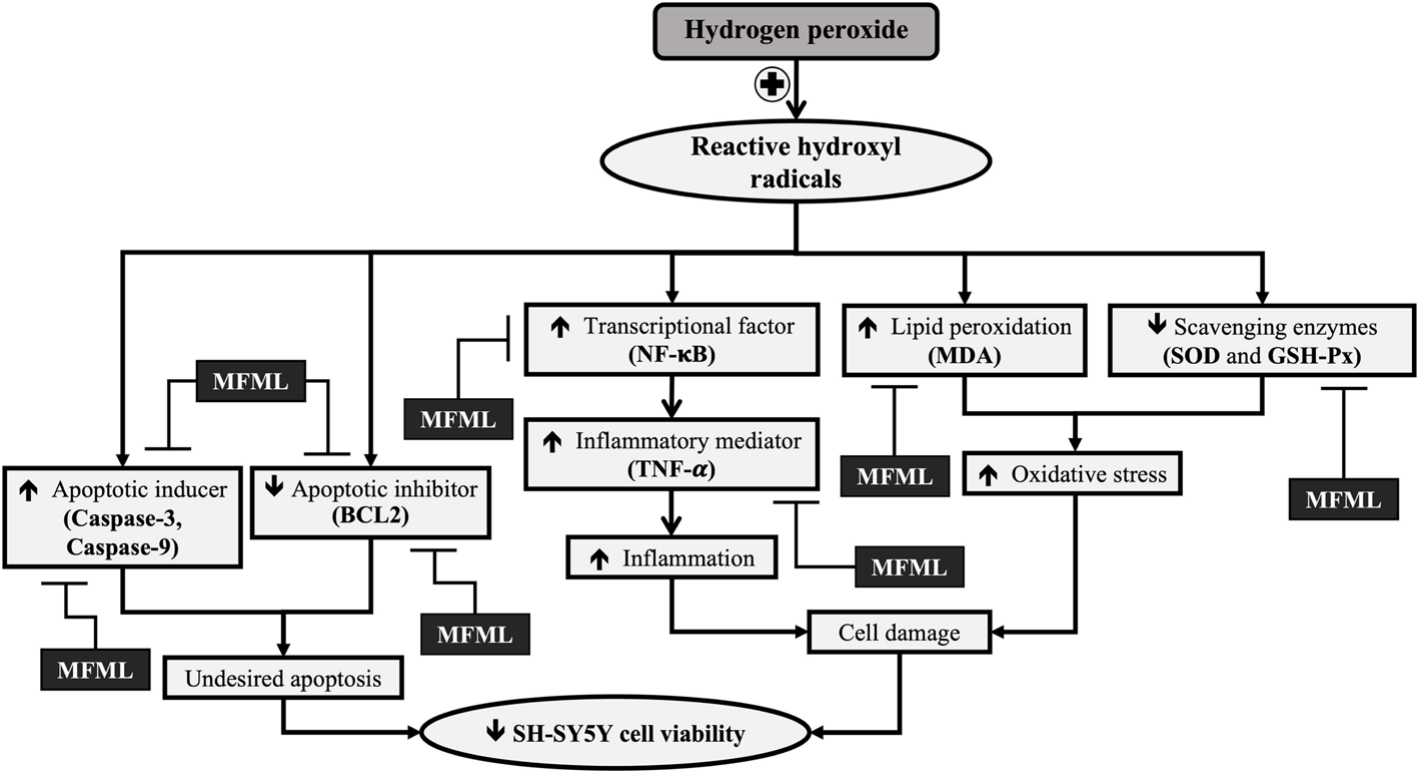
The schematic diagram demonstrated the neuroprotective effect of MFML in the hydrogen peroxide induced SH-SY5Y cytotoxicity. MFML: the combined extract of mulberry fruit and mulberry leaf; NF-κB: nuclear factor kappa-B; TNF-α: tumor necrosis factor- alpha; MDA: malondialdehyde; SOD: superoxide dismutase; CAT: catalase; GSH-Px: glutathione peroxidase

This study is the first study to demonstrate the neuroprotective effects of MFML. The possible underlying mechanisms might partly be through multitargets, including the improvement of oxidative stress, inflammation, and undesired apoptosis as shown in Fig 9. Therefore, MFML can serve as functional ingredients for developing neuroprotectants in neurodegenerative conditions. However, toxicity study is required in order to assure the safety of MFML consumption before moving forward to preclinical and clinical studies.

## Supplementary Information

**Additional file 1:** **Figure 4.** The expression of NF-κB in the hydrogen peroxide induced SH-SY5Y cell toxicity was detected by Western blotting. (1) naïve control, (2) H_2_O_2_ + vehicle, (3) H_2_O_2_ + MFML low dose, (4) H_2_O_2_ + MFML high dose. **Figure 5.** The expression of TNF-α in the hydrogen peroxide induced SH-SY5Y cell toxicity was detected by Western blotting. (1) naïve control, (2) H_2_O_2_ + vehicle, (3) H_2_O_2_ + MFML low dose, (4) H_2_O_2_ + MFML high dose. **Figure 6.** The expression of BCL-2 in the hydrogen peroxide induced SH-SY5Y cell toxicity was detected by Western blotting. (1) naïve control, (2) H_2_O_2_ + vehicle, (3) H_2_O_2_ + MFML low dose, (4) H_2_O_2_ + MFML high dose. **Figure 7.** The expression of Caspase-3 in the hydrogen peroxide induced SH-SY5Y cell toxicity was detected by Western blotting. (1) naïve control, (2) H_2_O_2_ + vehicle, (3) H_2_O_2_ + MFML low dose, (4) H_2_O_2_ + MFML high dose. **Figure 8.** The expression of Caspase-9 in the hydrogen peroxide induced SH-SY5Y cell toxicity was detected by Western blotting. (1) naïve control, (2) H_2_O_2_ + vehicle, (3) H_2_O_2_ + MFML low dose, (4) H_2_O_2_ + MFML high dose.

## Data Availability

The datasets generated during and/or analyzed during the current study are available from the corresponding author on reasonable request.
