# Supplementary material for: The neuroprotective effects of the combined extract of mulberry fruit and mulberry leaf against hydrogen peroxide-induced cytotoxicity in SH-SY5Y Cells
Source: BMC Complement Med Ther. 2023 Apr 13;23:117. doi: 10.1186/s12906-023-03930-z (PMC10100183; doi:10.1186/s12906-023-03930-z)
Supplement: Supplementary file 1 — Additional file 1: Figure 4. The expression of NF-κB in the hydrogen peroxide induced SH-SY5Y cell toxicity was detected by Western blotting. (1) naïve control, (2) H2O2 + vehicle, (3) H2O2 + MFML low dose, (4) H2O2 + MFML high dose. Figure 5. The expression of TNF-α in the hydrogen peroxide induced SH-SY5Y cell toxicity was detected by Western blotting. (1) naïve control, (2) H2O2 + vehicle, (3) H2O2 + MFML low dose, (4) H2O2 + MFML high dose. Figure 6. The expression of BCL-2 in the hydrogen peroxide induced SH-SY5Y cell toxicity was detected by Western blotting. (1) naïve control, (2) H2O2 + vehicle, (3) H2O2 + MFML low dose, (4) H2O2 + MFML high dose. Figure 7. The expression of Caspase-3 in the hydrogen peroxide induced SH-SY5Y cell toxicity was detected by Western blotting. (1) naïve control, (2) H2O2 + vehicle, (3) H2O2 + MFML low dose, (4) H2O2 + MFML high dose. Figure 8. The expression of Caspase-9 in the hydrogen peroxide induced SH-SY5Y cell toxicity was detected by Western blotting. (1) naïve control, (2) H2O2 + vehicle, (3) H2O2 + MFML low dose, (4) H2O2 + MFML high dose. [file 12906_2023_3930_MOESM1_ESM.pdf]

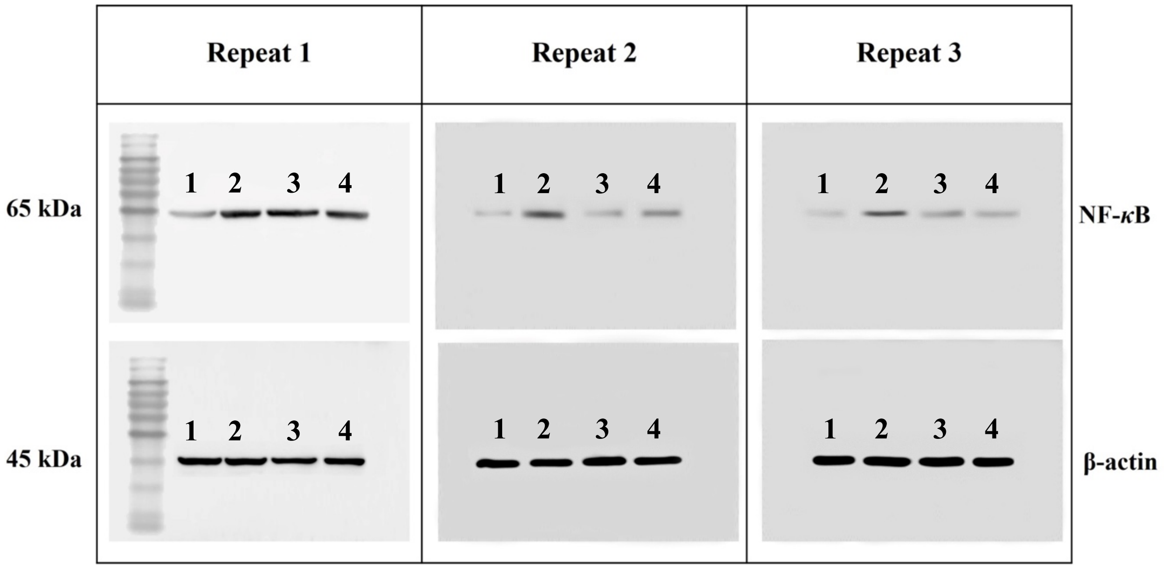

Figure 4: The expression of NF-κB in the hydrogen peroxide induced SH-SY5Y cell toxicity was detected by Western blotting**.** (1) naïve control, (2) H_2O_2 + vehicle, (3) H_2O_2 + MFML low dose, (4) H_2O_2 + MFML high dose.

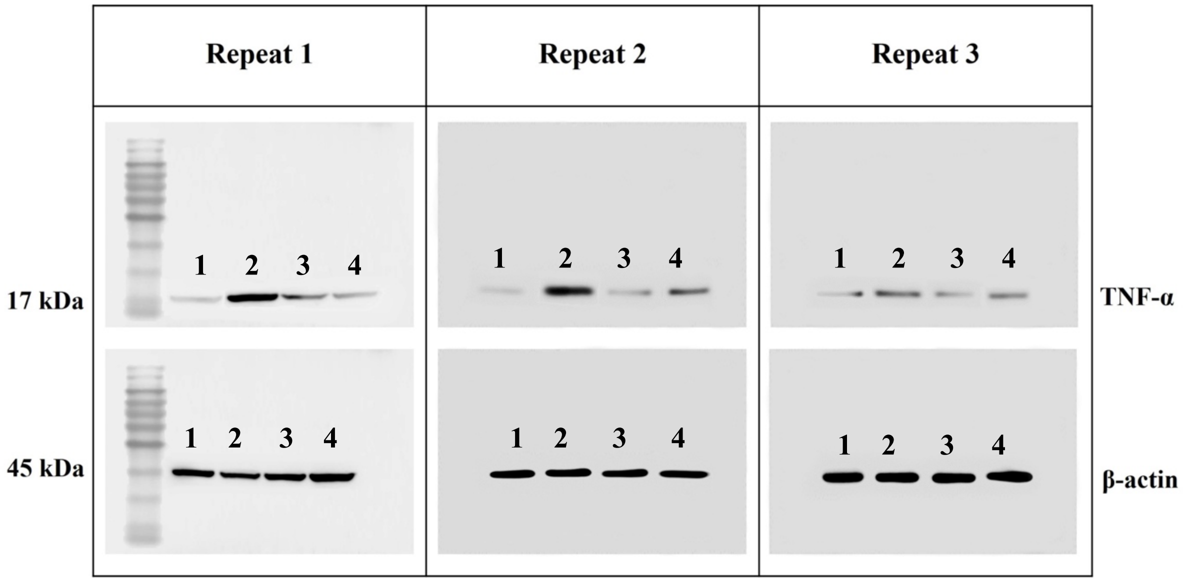

Figure 5: The expression of TNF-$\boldsymbol{\alpha}$ in the hydrogen peroxide induced SH-SY5Y cell toxicity was detected by Western blotting**.** (1) naïve control, (2) H_2O_2 + vehicle, (3) H_2O_2 + MFML low dose, (4) H_2O_2 + MFML high dose.

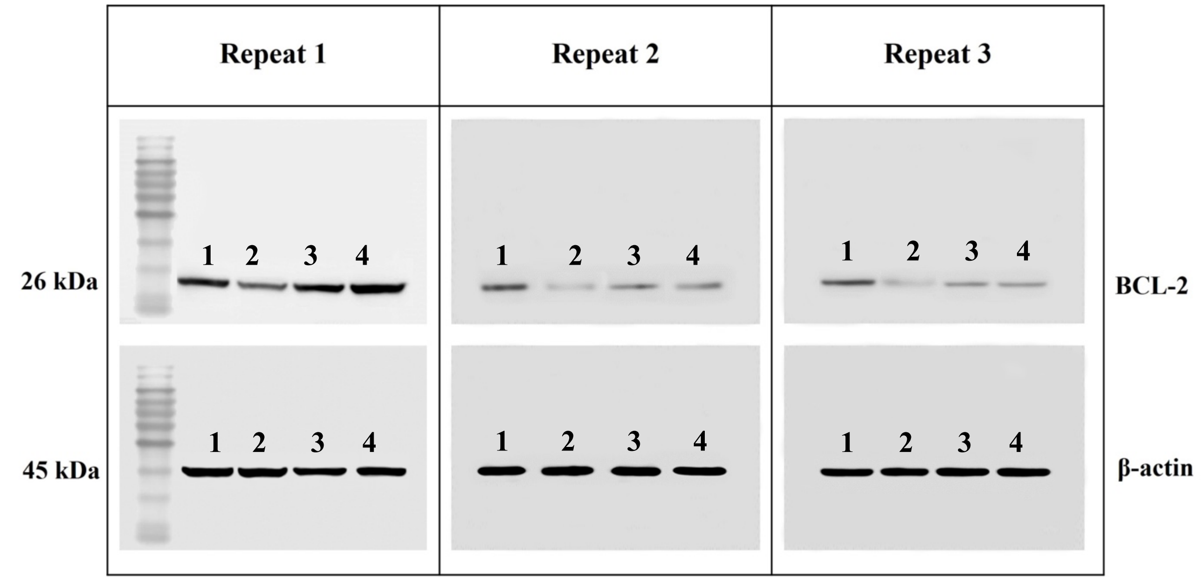

Figure 6: The expression of BCL-2 in the hydrogen peroxide induced SH-SY5Y cell toxicity was detected by Western blotting**.** (1) naïve control, (2) H_2O_2 + vehicle, (3) H_2O_2 + MFML low dose, (4) H_2O_2 + MFML high dose.

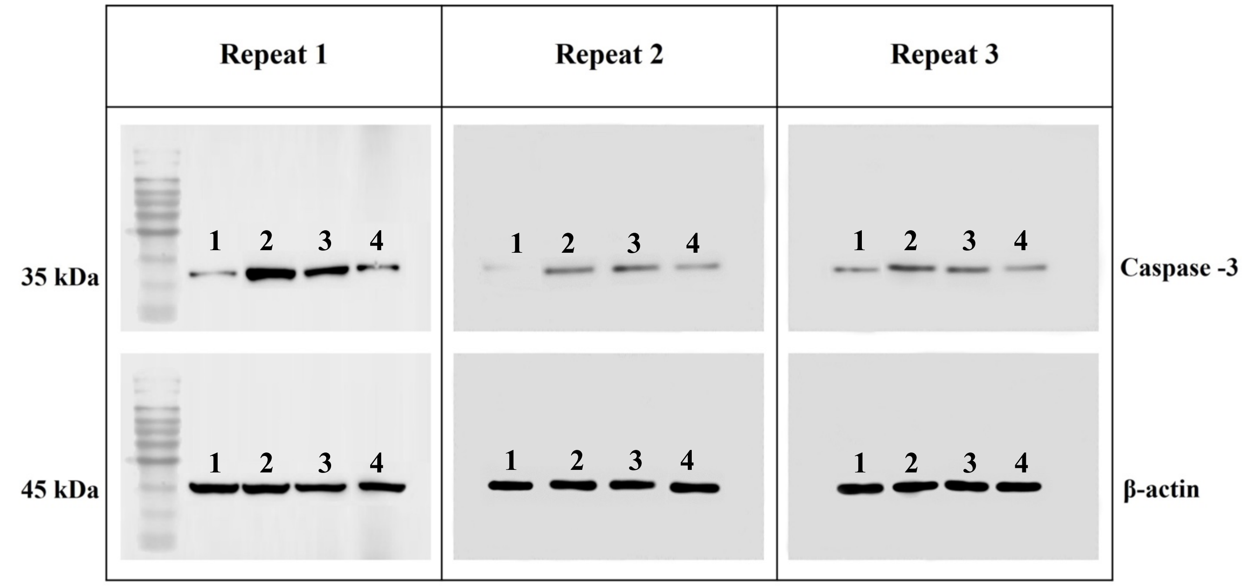

Figure 7: The expression of Caspase-3 in the hydrogen peroxide induced SH-SY5Y cell toxicity was detected by Western blotting**.** (1) naïve control, (2) H_2O_2 + vehicle, (3) H_2O_2 + MFML low dose, (4) H_2O_2 + MFML high dose.

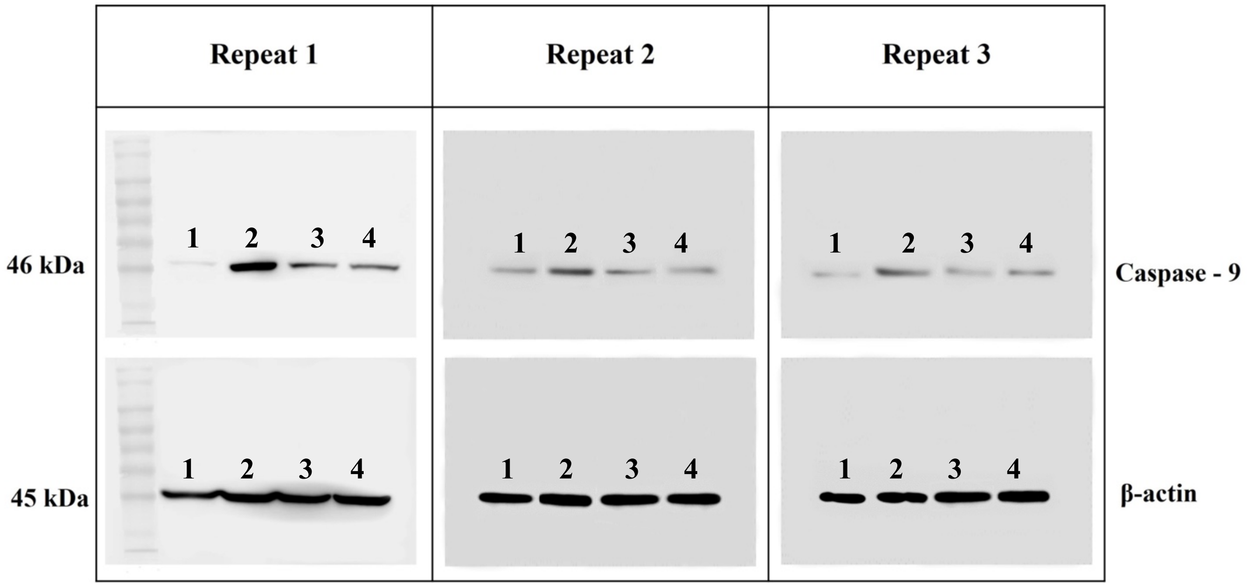

Figure 8: The expression of Caspase-9 in the hydrogen peroxide induced SH-SY5Y cell toxicity was detected by Western blotting**.** (1) naïve control, (2) H_2O_2 + vehicle, (3) H_2O_2 + MFML low dose, (4) H_2O_2 + MFML high dose.
